# Effects of a Web-Based Weight Loss Program on the Healthy Eating Index-NVS in Adults with Overweight or Obesity and the Association with Dietary, Anthropometric and Cardiometabolic Variables: A Randomized Controlled Clinical Trial

**DOI:** 10.3390/nu15010007

**Published:** 2022-12-20

**Authors:** Jan Kohl, Judith Brame, Pascal Hauff, Ramona Wurst, Matthias Sehlbrede, Urs Alexander Fichtner, Christoph Armbruster, Iris Tinsel, Phillip Maiwald, Erik Farin-Glattacker, Reinhard Fuchs, Albert Gollhofer, Daniel König

**Affiliations:** 1 Department of Sport and Sport Science, University of Freiburg, 79117 Freiburg, Germany; 2 Section of Health Care Research and Rehabilitation Research (SEVERA), Medical Center-University of Freiburg, Faculty of Medicine, University of Freiburg, 79106 Freiburg, Germany; 3 Department of Sport Science, Institute for Nutrition, Exercise and Health, University of Vienna, 1150 Vienna, Austria; 4 Department of Nutritional Sciences, Institute for Nutrition, Exercise and Health, University of Vienna, 1090 Vienna, Austria

**Keywords:** dietary quality, weight loss, cardiometabolic risk factors, body composition, dietary energy density, web-based intervention, fully automated, overweight, obesity

## Abstract

This randomized, controlled clinical trial examined the impact of a web-based weight loss intervention on diet quality. Furthermore, it was investigated whether corresponding changes in diet quality were associated with changes in measures of cardiovascular risk profile. Individuals with a body mass index (BMI) of 27.5 to 34.9 kg/m^2^ and an age of 18 to 65 y were assigned to either an interactive and fully automated web-based weight loss program focusing on dietary energy density (intervention) or a non-interactive web-based weight loss program (control). Examinations were performed at baseline (t0), after the 12-week web-based intervention (t1), and after an additional 6 (t2) and 12 months (t3). Based on a dietary record, the Healthy Eating Index-NVS (HEI-NVS) was calculated and analyzed using a robust linear mixed model. In addition, bootstrapped correlations were performed independently of study group to examine associations between change in HEI-NVS and change in dietary, anthropometric, and cardiometabolic variables. A total of *n* = 153 participants with a mean BMI of 30.71 kg/m^2^ (SD 2.13) and an average age of 48.92 y (SD 11.17) were included in the study. HEI-NVS improved significantly in the intervention group from baseline (t0) to t2 (*p* = 0.003) and to t3 (*p* = 0.037), whereby the course was significantly different up to t2 (*p* = 0.013) and not significantly different up to t3 (*p* = 0.054) compared to the control group. Independent of study group, there was a significant negative association between change in HEI-NVS and dietary energy density. A higher total score in HEI-NVS did not correlate with improvements in cardiovascular risk profile. The interactive and fully automated web-based weight loss program improved diet quality. Independent of study group, changes in HEI-NVS correlated with changes in energy density, but there was no association between improvements in HEI-NVS and improvements in cardiovascular risk profile.

## 1. Introduction

A high-quality diet, together with adequate physical activity, is a cornerstone in the prevention and treatment of overweight or obesity and related non-communicable diseases such as cardiovascular disease, cancer or type 2 diabetes [1]. Thus, on the one hand, the increasing sedentary lifestyle has crucial negative health effects [2]. On the other hand, the excessive consumption of high energy density foods rich in sugar and fat, such as sweets, high-fat meat or cheese, has been shown to promote higher energy intake, weight gain and the risk of overweight and obesity [3,4,5,6]. It has been suggested that lowering dietary energy density, in addition to reducing dietary quantity [7,8], may also have a positive impact on diet quality [9,10,11]. A central role of a high-quality and low-energy-dense diet is the consumption of fruits and vegetables, which, with their low energy density and high amount of fiber, can make an important contribution to satiety and the supply of essential micronutrients [8,12]. In this regard, a high intake of fruits and vegetables is associated with a lower risk of cardiovascular disease, cancer, and all-cause mortality in observational studies [13,14]. Nevertheless, only few individuals meet the national recommendations for their intake. In Germany, according to the National Nutrition Survey II (NVS II), 87.4% of those examined fall below the 400 g recommendation for daily vegetable intake of the German Nutrition Society (DGE) and 59% of the people did not reach the recommendation of 250 g fruit per day [15,16].

A balanced diet according to the recommendations of the DGE [17], the Dietary Guidelines for Americans [18] or the Mediterranean diet [19] with sufficient intake of fruit, vegetables, protein dairy products, fish and whole grains as well as moderation in spreadable fats, alcohol and meat should prevent overweight and non-communicable diseases [1,20,21]. Due to the multidimensionality of health aspects in nutrition, the Healthy Eating Index (HEI) is a useful tool to evaluate nutrition in its entirety. At the same time, a HEI allows assessment of whether dietary patterns are consistent with dietary recommendations. With regard to the DGE recommendations, the HEI-NVS [17,22] was developed based on the HEI-1995 [23] and HEI-EPIC [24], to assess whether dietary patterns are consistent with national recommendations. Studies on this are relevant because, in addition to weight loss in overweight and obesity, a healthy diet allows direct beneficial effects, through bioactive substances such as unsaturated fats, phytochemicals, fiber or micronutrients [25,26,27] and should therefore be additionally evaluated as part of a nutritional intervention.

Dietary quality indices such as the HEI are commonly used in cross-sectional and observational studies to examine associations between scores and various health outcomes or parameters. However, in order to evaluate the effectiveness of a dietary intervention, its use is also becoming increasingly important in intervention studies to assess the quality of nutrition over the course of an intervention [28,29]. While the association between diet quality indices and anthropometric or cardiometabolic variables has been well studied in cross-sectional studies [30,31,32] as well as the health outcomes in long-term cohort studies [1], the health-related effects of diet quality changes have been less well studied in comparatively short-term intervention studies. Limited evidence suggests that behavioral weight loss interventions can improve diet quality [29]. Whether changes in a diet quality index are associated with changes in cardiometabolic, anthropometric or other dietary variables during an intervention is sparsely studied.

The results of the NVS II showed that adherence to national nutrition recommendations in Germany, surveyed using the HEI-NVS, was low. On average, men had 67 and women 69 out of a possible 110 points [33]. Experience has demonstrated that interventions with a high reach and long duration are needed to support long-term behavior change [34]. Web-based interventions could provide a cost-effective alternative to face-to-face programs and meet outreach and accessibility requirements [35,36,37], but according to recent reports on fitness trends from the American College of Sports Medicine, the popularity of such web-based interventions is still comparatively low [38,39]. Increased technical capabilities and a more robust scientific base mean that web-based interventions are becoming more interactive and tailored, which improves the effectiveness [40]. Emerging evidence suggests that web-based interventions can promote healthy eating behavior [41,42,43,44], while studies failed to show significant effects during a web-based weight loss intervention [45]. Therefore, further research is needed to examine the interplay of web-based interventions for weight loss on diet quality and whether changes in dietary quality are associated with changes in other nutritional or physiological variables.

This intervention study aims to evaluate the effects of two different web-based weight loss programs on diet quality assessed by the HEI-NVS. The intervention group received a fully automated and interactive web-based weight loss program focusing on dietary energy density, while the control group was exposed to a non-interactive web-based weight loss program (informative website) which addressed the same topics. We hypothesize that the interactive web-based weight loss program would have a statistically significant positive effect on HEI-NVS and that this effect would be significantly greater than in non-interactive web-based weight loss program. Furthermore, this analysis will examine whether, independent of group allocation, changes in HEI-NVS are associated with changes in energy density, energy intake, anthropometric or cardiometabolic variables. This manuscript was prepared according to the CONSORT-EHEALTH checklist (File S1).

## 2. Materials and Methods

### 2.1. Study Design

This randomized controlled clinical trial contained two groups running in parallel, which were allocated by permuted block randomization in a 1-to-1 ratio [46]. Participants in the online questionnaire study, which examined German-language web-based weight loss programs independent of location, were eligible to participate in this clinical substudy if they resided in southwestern Germany (postal code beginning with 79). In this clinical study, participants were invited to the Department of Sport and Sports Science and underwent medical examinations. In addition to medical variables, the dietary and physical activity behavior of the participants was investigated. All variables were collected at baseline (t0), after the 12-week web-based intervention (t1), and after additional 6 (t2) and 12-month (t3) follow-up.

### 2.2. Participants and Recruitment

Participants in the online questionnaire study were notified of the opportunity to take part in the clinical substudy after enrollment if they provided the place of residence with postal code beginning with 79 [46,47]. For the clinical trial, people of any gender, age between 18 and 65 years, and body mass index from 27.5 to 34.9 kg/m^2^ were eligible to participate. Reasons for exclusion were breastfeeding or pregnancy as well as health problems or diseases. If existing health problems did not speak against participation in the program, this had to be certified with a medical certificate. Since the registration for the online questionnaire study as well as the registration for the clinical substudy took place online, appropriate computer skills were necessary. These were also required to use the web-based programs.

Various print and online media were used to recruit subjects for the clinical trial. Before study participants of southwestern Germany could register in the clinical substudy, they received the information on the study and had to provide written informed consent. After successful registration, randomization in the clinical substudy took place. In the subsequent telephone screening, potential study participants were again informed in detail about the study and the inclusion and exclusion criteria were reviewed. If the inclusion and exclusion criteria were not violated, an appointment was made for the baseline examination (t0). There, the final review of the criteria took place. Study participants received the Fitbit Charge 3 activity tracker (Fitbit, Inc.; San Francisco, CA, USA) as an incentive, which served as a measurement tool to record physical activity. Detailed information on participants and recruitment can be found in the study protocol [46].

### 2.3. Intervention

The intervention group’s interactive web-based program was divided into three sections. In the first section, diet could be documented and appropriate feedback was provided in terms of energy density, energy intake, and macronutrients. In addition, various activities could be selected to pursue personal goals. These activities were aimed at making the diet healthier, reducing energy intake and increasing physical activity. If an activity was selected, it was scheduled accordingly in the personal calendar.

The second section included evidence-based information on energy density, weight loss, and healthy eating. Topics were divided into articles and some were part of weekly tasks. The third area included personal statistics and feedback. Through this section, the own progress could be monitored.

In contrast, the control group received a non-interactive web-based program that covered the same topics by means of pure knowledge transfer. The information was divided into short articles, but there was no algorithm-controlled feedback and the diet could not be recorded. A detailed description of the intervention can be found in the study protocol [46].

### 2.4. Outcome

A seven-day dietary record, which was to be maintained at all measurement time points, was used to calculate HEI-NVS [46]. The HEI-NVS consists of 10 components (fruits, vegetables, grains, milk, meat, fish, eggs, spreadable fats, beverages and alcohol) and allows a maximum of 110 points. The components and scoring standards of the HEI-NVS can be found in Table S1 based on Wittig and Hoffmann [22]. While a maximum score of 15 is possible for the fruits and vegetables components, 10 points are possible for the remaining 8 components. Dietary records were obtained using the nutritional software NutriGuide Plus (Version 4.8, Nutri-Science GmbH; Freiburg, Germany). The logged food entries were assigned to the different components according to the logic of the HEI-NVS and the score for each component was calculated. The total HEI-NVS score was calculated from the sum of the component scores. According to the logic of the HEI, a higher score represents a healthier diet and a diet closer to the recommendations of the DGE. Thus, the full HEI-NVS score of 110 corresponds to a dietary behavior within the recommendations of the DGE.

In addition to dietary data, anthropometric and cardiometabolic variables were collected [46]. Body weight, fat mass, fat free mass and body height were analyzed with the validated bioelectrical impedance analysis scale Seca mBCA 515 [48,49,50] and the stadiometer Seca 274 (Seca GmbH & Co. KG; Hamburg, Germany). In addition, the waist circumference was measured with the Seca 201 (Seca GmbH & Co. KG; Hamburg, Germany) measuring tape. Study staff took standardized measurements between the lowest rib and the iliac crest [46]. Blood pressure was assessed using a clinically validated device (Boso Medicus Exclusive, BOSCH + SOHN GmbH & Co. KG; Jungingen, Germany). Furthermore, blood samples were taken and analyzed by the Clotten Medical Care Center (MVZ) in Freiburg. Blood lipids (total cholesterol, HDL cholesterol, LDL cholesterol), blood glucose (fasting blood glucose, HbA1c) and other variables not relevant in this analysis were collected. A detailed description of the measurements and outcomes has been described elsewhere [46].

### 2.5. Sample Size, Randomization and Blinding

Sample size was calculated using the primary outcome of body weight with an estimated dropout rate of 15%. The calculation resulted in a sample size of 150 (75 + 75) participants. Participants were randomly assigned to the two interventions in a 1:1 allocation ratio using permuted block randomization with variable blocks. The allocation sequence was generated by the Section of Health Care Research and Rehabilitation Research of the University Freiburg (SEVERA) using RITA software (version 1.50, University of Lübeck; Lübeck, Germany). Allocation of subjects was automated upon their registration for the study.

Because subjects could figure out their allocated program based on study information, blinding of subjects was not possible. Outcome assessors were blinded, whereas data analysts were not. Details on sample size calculation, randomization and blinding have been described elsewhere [46,47].

### 2.6. Data Analysis

All statistical analyses were performed using R (Version 4.1.3) and RStudio (Version 2021.09.1). Two analyses were conducted. First, a per protocol analysis (PP analysis) was performed with the complete cases (cases without missing values). Second, an intention-to-treat analysis (ITT analysis) was carried out using multiple imputation (in total 50 imputations), with all randomized cases included. For multiple imputation, the R package micemd [51] was used. In both analyses, the total HEI-NVS score was analyzed with a robust linear mixed model and a significance level at 0.05. The R packages lme4 [52] and robustlmm [53] were used for this purpose. Visualization of the descriptive results was performed using the R package ggplot2 [54]. Because the results of the PP and ITT analyses were comparable, only the ITT analysis is presented here, which is the primary analysis according to the CONSORT-EHEALTH checklist.

To examine the association between changes in HEI-NVS and changes in dietary, anthropometric and cardiometabolic variables independent of group, bootstrapped Pearson correlation was performed and a biased corrected 95% confidence interval calculated using the R package boot [55]. For this purpose, the difference of the corresponding variables of t1 minus t0 as well as t3 minus t0 was calculated. A bootstrap sample size of 5000 was used to investigate associations in the imputed data (ITT analysis). Due to the imputed data set, all of the *n* = 153 subjects could be included and bootstrapping was performed with replacement to draw with *n* = 153 cases.

## 3. Results

### 3.1. Recruitment, Drop-Outs and Baseline Characteristics

**Figure 1:**
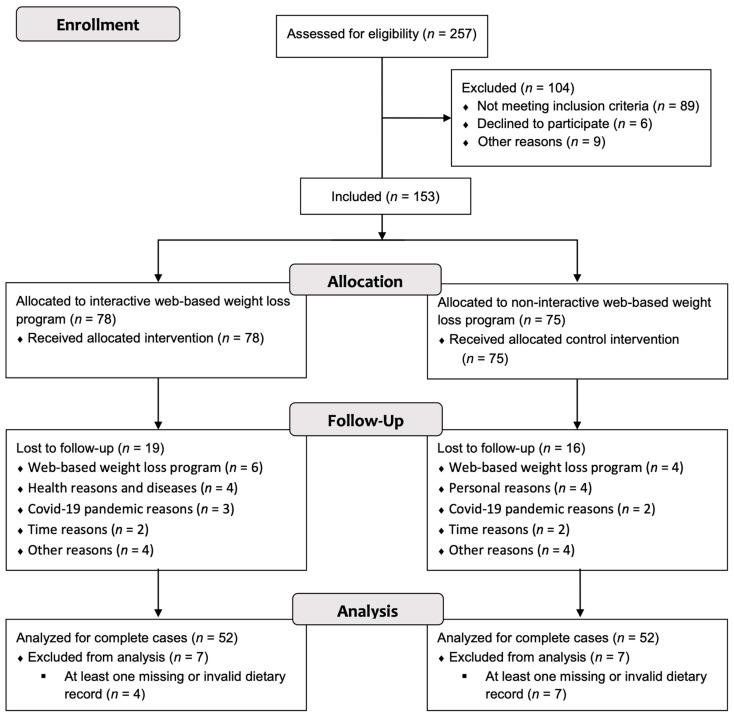
CONSORT flow chart depicting participant recruitment and drop-outs.

From January to July 2020, *n* = 257 interested individuals registered for the clinical substudy (Figure 1). Registered individuals were contacted by phone and checked for inclusion and exclusion criteria. If these criteria were not violated, the individuals were invited to the baseline examination, where a final screening of the criteria took place. After these screenings, *n* = 153 individuals successfully completed the baseline examination. During the course of the study, *n* = 35 (22.9%) dropouts were observed. In both groups, dietary data were available for *n* = 52 subjects each across all measurement time points. These *n* = 104 subjects could therefore be included in the PP analysis of the dietary data. The baseline characteristics of participants in the two study groups were consistently similar and are shown in Table 1.

### 3.2. Effects of Web-Based Weight Loss Programs on HEI-NVS

**Figure 2:**
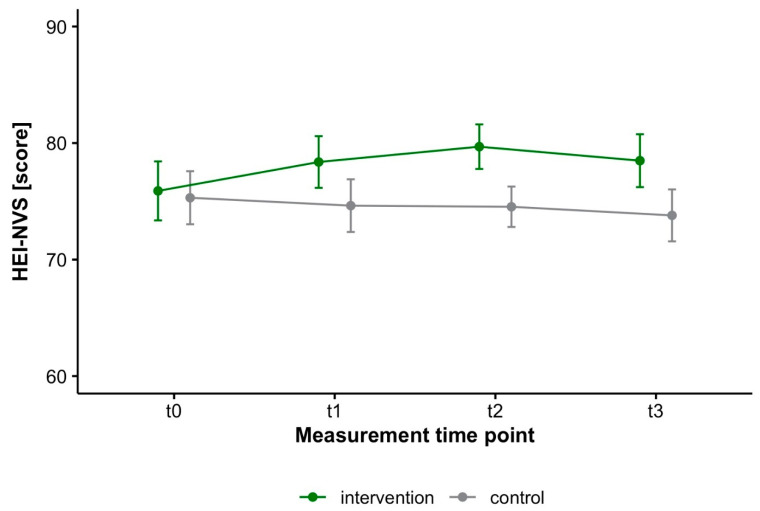
Mean and 95% confidence interval of the HEI-NVS for intervention (*n* = 78) and control (*n* = 75) (ITT analysis).

The total HEI-NVS score increased significantly within the intervention group from baseline (t0) to t2 and t3, but not to t1 (Figure 2 and Table 2. Descriptively, the intervention group improved over the course of the study, particularly in the vegetables, fish and meat component (Table 3). The increase of the total score corresponded to a small effect from baseline to every measurement timepoint (Table 4). Compared with the control group, which deteriorated slightly from a descriptive point of view (Table 4), the statistical analysis showed a significantly different course from baseline to t2 and non-significant to t1 and t3 (Table 2). An analysis on the effects of the web-based programs on weight loss can be found elsewhere [56].

### 3.3. Associations between HEI-NVS and Dietary, Anthropometric and Cardiometabolic Variables

The relationship between changes in HEI-NVS and other variables over the study period is presented in Table 5. Descriptive statistics of all variables used in the analysis can be found in Table S2. The changes in total HEI-NVS score correlated inversely with changes in energy density independent of group. Moreover, a weak positive correlation was observed between the change in HEI-NVS and the change in fasting blood glucose as well as a weak negative correlation with fat-free mass from t0 to t3. Apart from these findings, the analysis showed no further correlations between changes in HEI-NVS and other cardiovascular risk profile variables.

## 4. Discussion

The main finding of the present study was that a fully automated and interactive web-based health program focusing on the dietary energy density improved the total HEI-NVS score, thereby shifting participants’ diets toward the DGE dietary recommendations. Descriptively, these improvements were primarily due to improvement in the vegetables, fish, and meat components and resulted in a small effect in HEI-NVS from baseline to all three measurement timepoints. Compared to the non-interactive web-based weight loss program, however, there was only a significant advantage after 6-month follow-up (t0 to t2) and a non-significant difference after the 12-week intervention (t0 to t1) and after 12-month follow-up (t0 to t3). According to the meta-analysis published by Beleigoli and colleagues [45], none of the investigated web-based weight loss intervention demonstrated a significant advantage in diet quality over the control group. It should be noted that the included studies used very different instruments to measure dietary quality [45], which makes comparability difficult. Another meta-analysis on individuals with non-communicable diseases [44] showed benefits of eHealth interventions on healthy eating behaviors. The definition of healthy eating behaviors used in the studies included in this analysis had little overlap with diet quality. Thus, the outcomes used for inclusion were energy intake, macronutrient composition, and core food groups such as fruits or vegetables. While core food groups are often part of diet quality indices, diet quality is otherwise distinct from diet quantity and can only be represented to a limited extent, if at all, by macronutrient composition.

A recent systematic review demonstrated that weight loss interventions can improve diet quality as measured by a HEI [29]. Included studies covered in-person and mobile health interventions, which mostly resulted in an improvement between 4 to 7 points. In our study, mean improvements in the intervention group from baseline ranged from about 2.5 to 3.8 points, depending on the time of measurement. Thus, the improvement in this study tended to be lower than in the analysis by Cheng and colleagues [29]. However, it should be noted that the values cannot be directly compared because the review includes only studies using the U.S. versions HEI-2005, HEI-2010, and HEI-2015. These differ from each other and also from the HEI-NVS partly in components and evaluation system.

Besides the effect on the HEI-NVS of the web-based weight loss program focusing on reducing energy density, it was another important finding of the study that the change in HEI-NVS showed a weak to moderate inverse correlation with the change in energy density. An inverse relationship between energy density and diet quality has already been demonstrated in cross-sectional studies in various countries, e.g., Spain, Iran or Brazil [9,10,11], but to the best of our knowledge not yet in an intervention study. In contrast, improvements in HEI-NVS were not correlated with improvements in cardiovascular risk profile variables or changes in energy intake [57]. In contrast, the German National Nutrition Survey II, a representative cross-sectional study, found a positive association between HEI-NVS and BMI in women and in the 5th quintile in men [57]. In this longitudinal weight loss study, however, this was not confirmed with regard to body weight. Based on the absolute reference values of the HEI-NVS, it seems plausible that a higher score is associated with a higher energy intake and therefore a higher body weight or BMI. Also, with regard to the correlation between change in energy intake and HEI-NVS, this was not confirmed in this analysis.

Interestingly, this analysis revealed a weak positive correlation of change in HEI-NVS with change in fasting blood glucose as well as a weak negative correlation with change in fat-free mass. These results are surprising because it is assumed that improvement in a diet quality index is associated with better outcomes in cardiometabolic variables. There are several possible explanations for the lack of association between HEI-NVS and improvements in cardiometabolic variables. As previously reported, the observed effects on cardiometabolic variables by the two interventions were small. The effects on cardiometabolic variables have already been studied in the context of weight loss in people with overweight or obesity [58] and are consistent with the effects found in this study. Weight loss may already explain these effects, and the influence of diet quality may be insignificant and minor in the context of a weight loss intervention.

Furthermore, the construction of a diet quality index influences whether it correlates with diet quantity and thus with body weight and possibly other anthropometric variables. A negative association was shown between the change in HEI-NVS and the change in energy density, but not the change in energy intake. It is plausible that energy intake/dietary quantity and dietary quality may overlap if a diet quality index is not constructed appropriately. It is in fact possible that the HEI-NVS does not reflect diet quality independent of quantity. Due to the lack of reference to dietary quantity, as for example in the Healthy Eating Index-2015, a complete delineation to dietary quantity is compromised, as shown by the positive association between BMI and total score in women and partly in men [57]. Thus, the HEI-NVS measures compliance with the absolute amounts recommended in the German dietary guidelines rather than independent diet quality based on component reference values, which may tend to result in higher total scores if energy intake is high [57].

The following limitations must be considered when interpreting the results. First, the reference values of the HEI-NVS and the DGE, respectively, are based on systematic literature research and thus represent an important aspect for the preventive and therapeutic effects of nutrition in addition to the dietary quantity. However, considering other dietary indices and current findings in nutritional science, it is clear that important components of dietary quality such as sodium intake or carbohydrate quality such as intake of whole grains, refined grains, or added sugars are missing. The components and construction of the HEI-NVS may ultimately be responsible for the fact that improvements in HEI-NVS were not associated with improvements in cardiometabolic variables or, on the contrary, are partly even associated with negative effects on fasting blood glucose and fat-free mass. Thus, a differently constructed diet quality index might yield a different result.

Second, the reliability of self-reported data such as dietary records is limited. Recent data with reference data based on the doubly labeled water method suggests frequent underreporting, especially among people with overweight and obesity [59,60].

Third, compared to the real-world setting, both study groups may have been additionally motivated due to the activity tracker received as incentives as well as the free medical examinations provided by the study. In addition, complete blinding was not possible as subjects were likely to recognize their assigned program based on study information received in advance.

Fourth, the COVID-19 pandemic may also have influenced both groups in their dietary behavior. Because the COVID-19 pandemic and its limitations took a seasonal course, changes in dietary behavior are difficult to differentiate from seasonal changes and influence of the COVID-19 pandemic. The consumption of numerous food groups such as fruits, vegetables, or cereals, as well as energy intake, follows a seasonal pattern [61]. Simultaneously, an influence on dietary behavior could also be observed due to COVID-19 restrictions [62].

The elaborate implementation of seven-day dietary protocols is a strength of the present study. In addition, numerous anthropometric and cardiometabolic variables could be collected in a standardized manner in the clinical study. Therefore, this randomized controlled clinical trial provides more detailed insights into diet quality during web-based weight loss interventions and the association with dietary, anthropometric, and cardiometabolic variables.

## 5. Conclusions

Although the effect on HEI-NVS was small in the intervention group, this study demonstrated that a fully automated web-based weight loss intervention with focus on dietary energy density improved compliance with the national dietary recommendation. This result is relevant for all people aiming to reduce their body weight and eat healthier at the same time, but do not have access to personal care. Furthermore, the change in HEI-NVS showed an inverse correlation with the change in dietary energy density. Improvements in HEI-NVS were not associated with improvements in anthropometric and cardiometabolic variables. Interestingly, improvements in HEI-NVS were associated with only weak unfavorable effects on fat-free mass and fasting blood glucose. One might speculate that a diet quality index addressing other components, such as whole grains or salt, would have found more beneficial relationships. In the future, more intervention studies should address the association of diet quality and cardiovascular risk factors to examine the short-term effects of diet quality. This would provide a better understanding of the health effects of diet quality or related indices.

## Figures and Tables

**Table 1:** Baseline (t0) characteristics of the study participants ^1^.

Variables	All (*n* = 153)	Intervention (*n* = 78)	Control (*n* = 75)
Age [years]	48.92 (11.17)	49.12 (11.36)	48.72 (11.05)
Sex			
Male [n]	44 (28.8%)	20 (25.7%)	24 (32.0%)
Female [n]	109 (71.2%)	58 (74.3%)	51 (68.0%)
Body weight [kg]	88.39 (10.65)	88.42 (10.15)	88.36 (11.21)
Body height [m]	1.69 (0.08)	1.69 (0.07)	1.70 (0.08)
BMI [kg/m^2^]	30.71 (2.13)	30.88 (2.2)	30.54 (2.05)

^1^ Data are presented as mean (SD) and frequencies (%).

**Table 2:** Results of the robust linear mixed model of the HEI-NVS (ITT analysis) ^1^.

Predictors	HEI-NVS	*p*
Intercept	77.33 (2.66)	<0.001
Time		
t0–t1	5.23 (2.81)	0.063
t0–t2	9.06 (3.04)	0.003
t0–t3	5.90 (2.82)	0.037
Group (Control)	−1.10 (1.69)	0.513
Time * group (Control)		
t0–t1	−2.84 (1.79)	0.113
t0–t2	−4.96 (2.00)	0.013
t0–t3	−3.50 (1.81)	0.054

^1^ Unstandardized regression coefficients with standard errors in parentheses.

**Table 3:** Descriptive statistics of components of the HEI-NVS (ITT analysis) ^1^.

Group	t0	t1	t2	t3
Vegetables [score], max. 15 points				
Intervention	6.24 (3.41)	7.71 (4.06)	7.36 (3.01)	7.60 (3.51)
Control	6.15 (3.15)	6.67 (3.10)	5.72 (2.54)	6.57 (3.08)
Fruits [score], max. 15 points				
Intervention	7.95 (4.37)	7.89 (4.36)	7.75 (3.96)	7.34 (4.41)
Control	7.31 (4.69)	7.20 (4.63)	6.42 (3.61)	6.77 (3.99)
Grains [score], max. 10 points				
Intervention	6.67 (2.35)	6.42 (2.19)	6.98 (2.05)	6.56 (2.05)
Control	6.71 (2.33)	6.35 (2.04)	7.00 (1.94)	6.84 (2.30)
Dairy [score], max. 10 points				
Intervention	7.08 (2.15)	6.80 (1.95)	7.14 (1.75)	7.01 (2.05)
Control	7.15 (1.82)	7.33 (1.57)	7.27 (1.66)	7.14 (1.55)
Fish [score], max. 10 points				
Intervention	3.29 (3.90)	3.87 (3.85)	4.07 (3.36)	4.15 (3.39)
Control	4.31 (3.93)	3.18 (3.53)	3.52 (3.24)	3.13 (3.33)
Beverages [score], max. 10 points				
Intervention	8.93 (2.08)	8.97 (2.17)	8.87 (2.00)	8.75 (2.09)
Control	8.19 (2.52)	8.10 (2.84)	8.24 (2.48)	8.22 (2.59)
Eggs [score], max. 10 points				
Intervention	8.79 (1.92)	8.91 (1.82)	8.89 (1.62)	8.84 (1.75)
Control	8.72 (2.10)	8.31 (2.31)	8.90 (1.75)	8.62 (2.10)
Spreadable fats [score], max. 10 points				
Intervention	9.83 (0.82)	9.92 (0.43)	9.94 (0.37)	9.95 (0.22)
Control	9.76 (1.03)	9.91 (0.57)	9.93 (0.24)	9.90 (0.41)
Alcohol [score], max. 10 points				
Intervention	9.23 (1.79)	9.29 (1.54)	9.57 (1.17)	9.45 (1.17)
Control	9.04 (1.93)	9.11 (1.75)	9.30 (1.57)	9.18 (1.65)
Meat [score], max. 10 points				
Intervention	7.96 (2.43)	8.52 (1.98)	8.85 (1.51)	8.15 (1.94)
Control	7.95 (2.31)	8.13 (2.14)	8.08 (1.94)	7.83 (2.26)

^1^ Intervention (*n* = 78) and control (*n* = 75) over four measurement time points (t0: 0 months, t1: 3 months, t2: 6 months after t1, t3: 12 months after t1). Data are presented as mean values (SD).

**Table 4:** Effect sizes (Cohen’s d) with 95% confidence interval of the HEI-NVS (ITT analysis) ^1^.

Group	t0–t1	t0–t2	t0–t3
HEI-NVS			
Intervention	0.24 [−0.08, 0.55]	0.38 [0.06, 0.70]	0.24 [−0.07, 0.56]
Control	−0.07 [−0.39, 0.25]	−0.09 [−0.41, 0.23]	−0.15 [−0.48, 0.17]

^1^ Interpretation: |d| = 0.2: small effect, |d| = 0.5: medium effect, |d| = 0.8: large effect.

**Table 5:** Association between changes in HEI-NVS and changes in dietary, anthropometric, and cardiometabolic variables (ITT analysis) ^1^.

	Δt0–t1		Δt0–t3	
Variables	Correlation Coefficient	95% Confidence Interval	Correlation Coefficient	95% Confidence Interval
Energy density	−0.228 *	−0.359, −0.097	−0.312 *	−0.451, −0.165
Energy intake	0.089	−0.079, 0.256	0.076	−0.098, 0.247
Body weight	−0.052	−0.203, 0.122	−0.070	−0.235, 0.101
Waist circumference	0.068	−0.086, 0.216	−0.014	−0.203, 0.189
Fat mass	0.040	−0.103, 0.226	0.042	−0.111, 0.193
Fat free mass	−0.045	−0.209, 0.148	−0.190 *	−0.334, −0.041
Total cholesterol	−0.041	−0.185, 0.127	−0.018	−0.177, 0.133
HDL-cholesterol	−0.013	−0.165, 0.159	0.011	−0.163, 0.189
LDL-cholesterol	−0.087	−0.228, 0.065	0.001	−0.137, 0.151
Fasting blood glucose	−0.116	−0.258, 0.056	0.161 *	0.038, 0.275
HbA1c	−0.083	−0.217, 0.059	−0.055	−0.166, 0.081
Systolic blood pressure	0.104	−0.057, 0.264	−0.042	−0.221, 0.125
Diastolic blood pressure	0.176	−0.009, 0.365	−0.117	−0.297, 0.033

^1^ Bootstrapped Pearson correlation with biased corrected confidence interval. * Statistically significant correlation.

## Data Availability

The data of this study are the property of the Department of Sport and Sport Science of the University of Freiburg. The results of the clinical trials are presented to the scientific community and the trial sponsor as aggregated research data. Data on individual subjects cannot be reconstructed. No further data of the clinical trials are released to the scientific community or third parties.

## Supplementary Materials

The following supporting information can be downloaded at: https://www.mdpi.com/article/10.3390/nu15010007/s1, Table S1: Components and scoring standards of the HEI-NVS; Table S2: Descriptive statistics of variables used for correlation independent of study group. File S1: CONSORT-EHEALTH Checklist.
