# Supplementary material for: Effects of a Web-Based Weight Loss Program on the Healthy Eating Index-NVS in Adults with Overweight or Obesity and the Association with Dietary, Anthropometric and Cardiometabolic Variables: A Randomized Controlled Clinical Trial
Source: Nutrients. 2022 Dec 20;15(1):7. doi: 10.3390/nu15010007 (PMC9823428; doi:10.3390/nu15010007)
Supplement: Supplementary file 1 [file nutrients-15-00007-s001.zip › nutrients-2093978-supplementary.pdf]

nutrients

MDPI

Supplementary Materials

Table S1. Components and scoring standards of the HEI-NVS (adapted from [22]).

Component	Maximum points	Standard for 10 points/recommendation	Calculation
Fruits	15	250 g/day	Intake*10/recommendation
Vegetables	15	400 g/day	
Grains	10	350-560 g/day	If intake ≤ recommendation, then intake*10/recommendation<br>If intake > recommendation, then recommendation*10/intake
Dairy	10	2 portions<br>1 portion = 200-250 g of milk/yoghurt or 50-60 g of cheese/curd	
Fish	10	150-220 g/week	
Meat	10	< 300-600 g/week	If intake ≤ recommendation, then 10 points<br>If intake > recommendation, then recommendation*10/actual
Eggs	10	≤ 180 g/week	
Alcohol	10	women: ≤ 10 g ethanol/day<br>men: ≤ 20 g ethanol/day	
Spreadable Fats	10	≤ 15–30 g/day	
Beverages	10	≥ 1,5 l/day	If intake ≥ recommendation, then 10 points<br>If intake < recommendation, then intake*10/recommendation

Table S2. Descriptive statistics of variables used for correlation independent of study group (ITT analysis). [1]

Variables	t0	t1	t3
HEI-NVS [score]	75.61 (10.56)	76.54 (9.98)	76.19 (10.13)
Energy density [kcal/g]	1.64 (0.35)	1.48 (0.32)	1.55 (0.33)
Energy intake [kcal/day]	1978.1 (574.46)	1820.19 (499.69)	1865.35 (544.52)
Body weight [kg]	88.39 (10.65)	85.68 (10.81)	85.63 (11.41)
Waist circumference [cm]	100.42 (9.22)	98.04 (9.15)	97.15 (8.95)
Fat mass [kg]	35.09 (6.16)	33.05 (6.12)	33.40 (6.71)
Fat free mass [kg]	53.24 (9.19)	52.43 (9.04)	52.04 (8.54)
Total cholesterol [mg/dl]	215.22 (41.92)	209.30 (39.38)	209.04 (38.14)
HDL-cholesterol [mg/dl]	57.60 (11.50)	57.14 (10.10)	59.56 (10.63)
LDL-cholesterol [mg/dl]	135.00 (35.20)	131.42 (31.66)	142.18 (30.24)
Fasting blood glucose [mg/dl]	89.24 (10.04)	89.17 (8.95)	87.85 (11.00)
Hba1c [%]	5.41 (0.43)	5.40 (0.34)	5.46 (0.29)
Systolic blood pressure [mmHg]	129.58 (13.73)	126.63 (13.56)	127.37 (12.19)
Diastolic blood pressure [mmHg]	87.56 (8.77)	85.50 (8.70)	86.51 (7.77)

1 Data are presented as mean (SD).

References

22 Wittig, F.; Heuer, T.; Claupein, E.; Pfau, C.; Cordts, A.; Schulze, B.; Padilla Bravo, C.A.; Spiller, A. (Eds.) *Auswertung der Daten der Nationalen Verzehrsstudie II (NVS II): Eine Integrierte Verhaltens- und Lebensbasierte Analyse des Bio-Konsums*; Rubner-Institut: Karlsruhe, Germany; Georg-August-University Göttingen: Göttingen, Germany, 2010; pp. 51–68.
